# The prospective approach for aptamers applied in the treatment and molecular diagnostics of ischemic stroke

**DOI:** 10.3389/fphar.2025.1553337

**Published:** 2025-04-28

**Authors:** Wenfeng Li, Junyi Wu, Zijian Hu, Jixuan Zhang, Guangming Ye, Fengling Luo, Zhikun Zeng, Yi Luo

**Affiliations:** ^1^ Department of General Surgery, Liver Transplantation Centers, Huashan Hospital, Fudan University, Shanghai, China; ^2^ The Second Clinical College of Wuhan University, Wuhan, Hubei, China; ^3^ Peking University Huilongguan Clinical Medical School, Beijing Huilongguan Hospital, Beijing, China; ^4^ Department of Laboratory Medicine, Zhongnan Hospital of Wuhan University, Wuhan, Hubei, China; ^5^ Hubei Provincial Clinical Research Center for Molecular Diagnostics, Wuhan, Hubei, China; ^6^ State Key Laboratory of Virology and Hubei Province Key Laboratory of Allergy and Immunology, and Department of Immunology, Wuhan University Taikang Medical School (School of Basic Medical Sciences), Wuhan, Hubei, China

**Keywords:** aptamer, SELEX, ischemic stroke, central nervous system, targeted therapy, molecular diagnostics

## Abstract

Ischemic stroke is a leading cause of death and disability worldwide. Therefore, there is a critical need to explore the underlying mechanisms and develop effective treatment strategies for ischemic stroke. As small and non-immunogenic nucleic acid molecules, aptamers can be easily chemically modified, break through the blood-brain barrier, and be screened using the classic Systematic Evolution of Ligands by Exponential Enrichment. With the advancements in emerging technologies, aptamer-based strategies have provided diagnostic and therapeutic potential for applications in central nervous system diseases. Aptamers have become a useful tool for targeted therapy and biomarker discovery in ischemic stroke. This review presents recent advances and perspectives on aptamer applications in stroke prevention, treatment, and diagnosis, focusing on targeting pathological blood clotting or thrombosis, inflammatory responses, and specific biomarkers in key cells.

## 1 Introduction

Aptamers are a class of single-stranded DNA or RNA that can bind to various target molecules with high affinity and specificity, such as proteins, peptides, cells, pathogens, and viruses (Jayasena, 1999; Zhang et al., 2019). Aptamers can be screened directly from *in vitro* libraries using the classic Systematic Evolution of Ligands by Exponential Enrichment (SELEX) method (Tuerk and Gold, 1990). As an emerging recognition element in the construction of biosensors, aptamers have more advantages than traditional antibodies because they are smaller, have lower toxicity and immunogenicity, and are easier to synthesize (Toh et al., 2015; Zhuo et al., 2017). Thus, aptamers have attracted increasing attention for their clinical applications, especially in peripheral system diseases, such as cancer, and inflammatory diseases (Zhuo et al., 2017; Zhou et al., 2018). Aptamers can easily penetrate the blood-brain barrier (BBB), revealing their potential value in neurological diseases. Aptamer-based tests or biosensors targeting specific biomarkers may contribute to the diagnosis, therapy, and imaging of central nervous system (CNS) diseases (Ozturk et al., 2021; Qu et al., 2017).

Stroke is a leading cause of morbidity and disability worldwide. Ischemic stroke accounts for approximately 85% of all stroke cases and leads to the development of cerebral ischemia and ischemia-reperfusion injury (Katan and Luft, 2018). Rapid reperfusion via intravenous thrombolysis or endovascular thrombectomy is a common treatment for ischemic stroke. However, considering the narrow therapeutic window and safety concerns, current treatments do not provide maximal benefit and functional recovery in stroke patients (Mao et al., 2022). Brain ischemia can cause a complex series of pathophysiological events, including oxidative stress, inflammation, apoptosis, ionic imbalance, and excitotoxicity (Kuriakose and Xiao, 2020). The exploration of the underlying mechanisms and effective treatment strategies via potential targets in ischemic stroke should be a good approach. Because aptamers have the ability to bind specifically to proteins or cells, scientists are trying to use aptamers to treat brain ischemia or detect biomarkers that reflect pathological processes. This review mainly focuses on recent advances in aptamer-based applications in clinical medicine, with an emphasis on their applications in the treatment and molecular detection of ischemic stroke, and their future prospects.

## 2 How to screen specific aptamers

**FIGURE 1:**
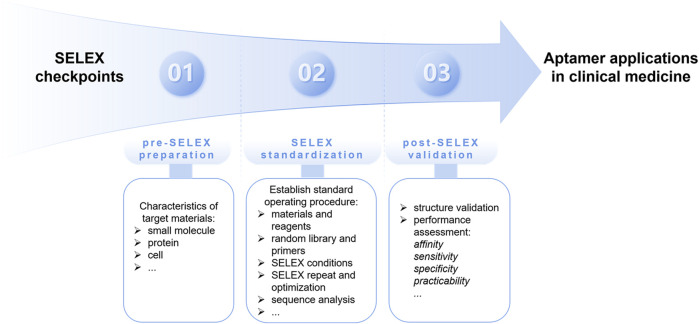
The proposed SELEX checkpoints for *de novo* aptamer selection, including pre-SELEX preparation, SELEX standardization, and post-SELEX validation.

SELEX is a combinatorial chemical technique used to screen specific aptamers from a large nucleic acid library containing various candidates. The main procedure of classical SELEX includes repeated rounds of partitioning, amplification, and selection with high affinity for targets (Tuerk and Gold, 1990). Since its generation in 1990, the SELEX technology has been continuously developed. To recognize different targets with their respective characteristics, a series of novel SELEX methods have been generated (Huang et al., 2021). Recently, minimum aptamer publication standards for *de novo* aptamer selection have been proposed, emphasizing standardization of the specificity and repeatability of the SELEX process (McKeague et al., 2022). The following checkpoints should be considered when screening and obtaining specific aptamers, including pre-SELEX preparation, SELEX standardization, and post-SELEX validation (Figure 1).

### 2.1 Pre-SELEX preparation

A full understanding of the characteristics of the target material is the first critical step in determining which SELEX method should be used, as the nature of different types of targets-ranging from small molecules to proteins and even whole cells-dictates the specific challenges and requirements for aptamer selection.

(i) Small molecular targets. Small molecules (with a relative molecular mass less than 1,000, such as toxins, antibiotics, and hormones) have simple structures that typically feature with few binding sites and weak affinities for nucleic acids. This makes it challenging to separate small molecule-nucleic acid complexes (Ruscito and DeRosa, 2016). However, with the rapid advancement of SELEX technology, several innovative methods have been developed to optimized the recognition, fixation, and separation processes, including capture-SELEX, graphene oxide-SELEX, GOLD-SELEX, capillary electrophoresis-SELEX, and affinity chromatography-SELEX (Zhang et al., 2019). Among these methods, capture-SELEX using magnetic beads has been specifically developed to immobilize oligonucleotides rather than the small molecule targets themselves. This approach offers several advantages, such as rapid separation, ease of modification, and the ability to work with low target amounts (Lauridsen et al., 2018). Additionally, graphene oxide-SELEX allows for the screening of small molecules in their natural state, and it can even be used to identify multi-target aptamers (Nguyen et al., 2014).
(ii) Protein targets. Most protein targets have large molecular weights and complex structures and properties; therefore, it may be difficult to plan the SELEX procedure with the purified soluble protein target directly (Bayat et al., 2018). For the target protein requiring the presence of co-receptor or location in cell membrane, the novel complex target SELEX can realize selective isolation of aptamers from various cell surface proteins (Shamah et al., 2008). Capillary electrophoresis-SELEX can be used for non-immobilized protein targets without steric hindrance (Yan et al., 2019). Atomic force microscopy-SELEX can isolate aptamers with high affinity by detecting the force between the proteins on the sample surface and oligonucleotide probes (Miyachi et al., 2010).
(iii) Cell targets. Cell-SELEX was recently developed to select target molecules or proteins in their native state, instead of tedious extraction and purification of targets. It is generally divided into two types: directly targeting cells and expressing targets in cells using gene editing (Duan et al., 2022). To exclude non-specific selection, suitable materials must be prepared for negative or counter selection. For the cell-based assay, normal or untreated cells are used as controls. Moreover, the *in vivo* distribution, concentration and analogs of target materials should be considered.

### 2.2 SELEX standardization

In traditional SELEX or its variants, multiple parameters may affect the discovery and function of high affinity aptamers. Thus, establishing a standard operating procedure (SOP) for SELEX is the basis for successful aptamer screening. The following elements should be included in the standard operating procedure. i) Materials and reagents. First, information on positive and negative/counter targets should be provided, because the screening result depends on the chosen target. The most common protein targets and their expression ranges, tags, expression systems, and amino acid sequences should be listed. Then, a detailed immobilization strategy for the target should be considered, including the conditions and type of immobilization, related agent sources and concentrations, constituents of the binding/selection/blocking buffer, and pH. ii) Random library and primers. The length of the random region and primer binding sites in the library are the key parameters for the SELEX procedure; therefore, the sequences of the library and primers should be listed. The following information is also useful: the size and concentration of the library and the synthesis and purification of the library. iii) SELEX conditions. Based on the library/target information and target immobilization, the subsequent SELEX process should be performed step-by-step, and a clear schematic illustration is recommended. Several key points need to be addressed, including partitioning conditions, selection conditions for each round, preparation of pools for each round, PCR conditions, and negative/counter selection. iv) SELEX repetition and optimization. Given that the published selection criteria will be adopted by other individual laboratories, all experiments should be repeated at least three times under similar conditions to ensure feasibility, and then optimized to the publishable standard. v) Sequence analysis. After the SELEX process is completed, the selected aptamer pool should be determined using high-throughput sequencing. The sequencing results were compared, homology of the aptamer sequences was analyzed, and aptamers with high proportions were selected for further experiments.

### 2.3 Post-SELEX validation

To obtain an excellent aptamer, further post-SELEX validation is necessary, including structural validation and performance assessment of affinity, sensitivity, specificity, and practicability. First, the selected candidates are used to predict the minimum free energy of the aptamer sequences and evaluate the stability of the secondary structure. The predicted stem-loop structure is one of the most commonly used constituents in target recognition. A lower minimum free energy and higher percentage of G/C may increase the stability of the aptamer-target complex. The equilibrium dissociation constant (*K*d) of the candidate aptamers is determined to evaluate the affinity for the target using several methods such as surface plasmon resonance analysis. The lowest *K*d value indicates the highest affinity, which is used as a key evidence for the superiority of the aptamer. Subsequently, molecular docking is applied to predict the binding sites and key interactions between the target and selected aptamer. The aptamer is optimized by further truncation to obtain the shortest possible length and an acceptable affinity. A specific aptasensor may be then designed to evaluate the sensitivity and specificity of the screened aptamer to achieve practicability.

## 3 Recent advances in aptamer applications for ischemic stroke

Owing to the aforementioned advantages and widely applicable targets, aptamers, also called chemical antibodies, show great potential in the diagnosis, therapy, and imaging of diseases in clinical settings. Aptamers can cross the BBB to recognize and conjugate to specific targets in the CNS, providing an opportunity for their application in neurological disorders such as ischemic stroke (Monaco et al., 2017). Aptamers have gradually become a useful tool for targeted therapy and biomarker discovery in ischemic stroke research.

### 3.1 Target to pathological blood clotting or thrombosis

Pathological blood clotting or thrombosis can limit vital blood flow to organs, leading to catastrophic events, including ischemic stroke. Recent studies have reported that aptamer systems can increase the efficacy and safety of antithrombotic and anti-platelet treatments, which may be the mainstay of stroke therapy or prevention (Henninger and Mayasi, 2019). Aptamers that target the von Willebrand Factor (vWF) against platelet function are of continuous interest. Under pathological conditions, the vWF is activated through physical deformation that exposes its A1 domain and enables its binding to the platelet receptor glycoprotein 1b, resulting in thrombosis. Attacking the interaction between the vWF and glycoprotein 1b on the outer shell of thrombus may facilitate recanalization of platelet-rich thrombotic occlusions (De Meyer et al., 2012). From the first aptamer ARC1172 against the vWF to the newest generation of BB-031/DTRI-031 aptamers, several vWF-related aptamers have been reported, some of which are in the clinical trial stage (Table 1) (Huang et al., 2009; Ismail et al., 2024; Gilbert et al., 2007; Markus et al., 2011; Siller-Matula et al., 2012; Zhu et al., 2020a; Zhu et al., 2020b; Kovacevic et al., 2021; Sakai et al., 2020; Nimjee et al., 2019; Shea et al., 2022; Carfora et al., 2024). As not systemic induction of fibrinolysis as does recombinant tissue plasminogen activator, anti-vWF aptamers would be a highly desirable alternative to current therapeutic approaches without the hemorrhage risk. However, increased peri-operative bleeding and anemia associated with ARC1779 have been reported in a previous randomized trial (Markus et al., 2011). One approach to mitigate the risk of hemorrhage is to use an aptamer inhibitor. As oligonucleotide sequences, aptamers provide a code for complementary sequences that can be used to inhibit their functions (Henninger and Mayasi, 2019). For example, the complementary antidote RNA aptamer Ch-9.14-T10 can reverse the vWF aptamer ARC1779 activity *in vitro* and *in vivo*, resulting in substantial attenuation of bleeding in surgically challenged mice (Nimjee et al., 2012). Similarly, a complementary aptamer, BT101, was developed to specifically reverse BT100- or BT200-induced effects on vWF activity and platelet function without any adverse effects (Zhu et al., 2020b). The aptamer-antidote system may realize the safety and efficacy of aptamers used for ischemic stroke prevention and therapy, in addition to targeting the vWF. One such system is REG1, which blocks the conversion of factor IX to IXa. In a phase I/II trial, controllable anticoagulation therapy was reported in healthy volunteers, without obvious bleeding or side effects. Nevertheless, in another phase III trial, REG1 was suggested to be associated with severe allergic reactions, and there was a lack of evidence that it reduced ischemic events or bleeding compared with bivalirudin (Zhu et al., 2020; Chan et al., 2008). Therefore, the positive and negative effects of REG1 require further investigation.

**TABLE 1:** Summary of aptamers targeting pathological blood clotting or thrombosis for stroke treatment.

Aptamer	Mode of action	Application	Developmental stage
ARC1172	Binds the A1 domain of vWF	Inhibits vWF-dependent platelet aggregation to prevent or treat thrombosis (Huang et al., 2009) ARC1172-based biotherapeutic conjugate for shear responsive release of vWF A1 domain (Ismail et al., 2024)	Preclinical (*in vitro*)
ARC1779	Blocks the interaction of vWF with GP1b on platelets	Inhibits platelet aggregation with less increase in bleeding than conventional antiplatelet agents (Gilbert et al., 2007) Reduces cerebral embolism after CEA in stroke patients (Markus et al., 2011)	Phase I/II
ARC15105	Fully 2′OME modified ARC1779	A potent antagonist of vWF-mediated platelet activation, aggregation and adhension (Siller-Matula et al., 2012)	Preclinical (*in vitro and* *in vivo*)
BT100	Four more extra base-pairs added to ARC15105 (Zhu et al., 2020a)	/	/
BT200	A pegylated form of the aptamer BT100	Promising inhibition of human vWF and prevention of arterial occlusion in cynomolgus monkeys (Zhu et al., 2020b) Blocks vWF and platelet function in blood of patients with LAA stroke (Kovacevic et al., 2021)	Preclinical (*in vitro and* *in vivo*) Phase I
TAGX-0004	To the vWF A1 domain	Shows total inhibition of thrombus formation superior to ARC1179 (Sakai et al., 2020)	Preclinical (*in vitro*)
DTRI-031	A vWF aptamer-antidote pair	A novel, rapidly reversible antiplatelet agent that may prove valuable for the treatment of acute thrombotic events in the heart, brain, and peripheral vasculature (Nimjee et al., 2019)	Preclinical (*in vitro and* *in vivo*)
BB-031 (also called DTRI-031)	Targets vWF A1 domain-platelet GP1b interactions	Dose-dependent vWF inhibition by BB-031 correlates with thrombolysis in a microfluidic model of arterial occlusion (Shea et al., 2022) Targeted inhibition of vWF by BB-031 increases recanalization and reperfusion, and reduced infarct volume in a canine model of BAO stroke (Carfora et al., 2024)	Preclinical (*in vitro and* *in vivo*)
REG1 (pegnivacogin, RB006 & anivamersen, RB007)	Blocks conversion of factor IX to IXa	Controllable anticoagulation in healthy volunteers without bleeding events or side effects (Chan et al., 2008) REG1 is associated with severe allergic reactions without reduction of ischemic events or bleeding compared with bivalirudin (Lincoff et al., 2016)	Phase I/IIb/III
ssDNA aptamer for uPA	Binds human pro-urokinase (Skrypina et al., 2004)	/	/
K18/K32	tPA-binding RNA aptamers inhibiting the tPA/LRP-1 complex formation	Inhibits tPA-LRP-1 association and LRP-mediated endocytosis in human astrocytes and vascular endothelial cells (Bjerregaard et al., 2015)	Preclinical (*in vitro*)
Ch-9.3t	Binds to factor IXa	Attenuate neurological function, reduce thrombin generation, and decrease inflammation in murine models of stroke (Blake et al., 2011)	Preclinical (*in vivo*)
AYA1809002/AYA1809004	Binds to the active site of thrombin	Suggests to be the potent anti-thrombin candidates (Ayass et al., 2023)	Preclinical (*in vitro*)

Note: vWF, von Willebrand factor; GP1b, glycoprotein 1b; CEA, cartoid endarterectomy; LAA, large artery atherosclerosis; BAO, basilar artery occlusion; uPA, urokinase-type plasminogen activator; tPA, tissue plasminogen activator; LRP, low density lipoprotein receptor related protein.

As early as 2004, single-stranded DNA aptamers that bind to human pro-urokinase were screened; however, further application research is required (Lincoff et al., 2016). With the emergence of tissue plasminogen activator (tPA) thrombolysis for ischemic stroke, minimization of tPA-mediated toxicity to optimize the thrombolytic effect has attracted increasing attention (Henninger and Mayasi, 2019). For example, tPA has several adverse effects mediated by its interaction with low-density lipoprotein receptor-related protein-1 (LRP-1) (Skrypina et al., 2004). Therefore, tPA-binding RNA aptamers have been developed to inhibit tPA/LRP-1 complex formation. Two aptamers (K18/K32) were found to efficiently inhibit tPA-LRP-1 association and LRP-mediated endocytosis in human astrocytes and vascular endothelial cells, providing a viable strategy to improve the safety of thrombolytic treatment in stroke through co-administration with tPA (Yepes, 2024). Additionally, a factor IXa aptamer, Ch-9.3t, administered intravenously in murine models of stroke can attenuate neurological function, reduce thrombin generation, and decrease inflammation (Bjerregaard et al., 2015). The two most potent aptamers, AYA1809002 and AYA1809004, which bind to the active site of thrombin, were recently suggested as potent anti-thrombin candidates (Blake et al., 2011). Further studies are necessary to validate the safety and efficacy of these aptamers in clinical settings.

### 3.2 Target to inflammatory responses

The onset of stroke can initiate an inflammatory cascade in both the CNS and systemic immune system, which plays a crucial role in the progression of ischemic pathology (Ayass et al., 2023). Therefore, aptamers targeting inflammatory responses may be helpful tools for stroke treatment and molecular diagnosis (Table 2). The detection of C-reactive protein (CRP) could significantly predict a population with high risk of stroke. Patented CRP aptamers have been developed as biomolecules for detection and experimental assays of CRP inhibition (Guo et al., 2023). These aptamers may contribute to the design of aptamer-dependent drugs and delivery strategies for future applications (Miramontes-Espino and Romero-Prado, 2013). Anaphylatoxin complement component 5a (C5a) is a potent inflammatory mediator generated during complement activation and is implicated in inflammatory and neuronal damage. NOX-D20, an active L-oligonucleotide aptamer that can bind to the physiological sites in C5a to block its binding to CD88, was screened *in vitro* (Wu et al., 2016). Furthermore, a framework nucleic acidconjugated with anti-C5a aptamers was used for stroke treatment in a rat model and showed rapid penetration into different brain regions and effective alleviation of neurotoxicity and inflammation in the brain (Yatime et al., 2015). Here, the development of toll-like receptor 4 (TLR4)-binding aptamers for the treatment of ischemic stroke needs to be mentioned. TLR4 plays a fundamental role in the activation of innate immunity and inflammatory response elicited by ischemic insults (Li et al., 2019). Since TLR4 mediates brain damage, screening for TLR4-blocking aptamers may be a useful strategy for the treatment of stroke. In two recent studies, a truncated form of ApTLR#4FT, ApTOLL, showed long-lasting protective effects against brain damage induced by middle cerebral artery occlusion in rats, supporting its promising application in patients undergoing arterial recanalization (Oo, 2024; Fernandez et al., 2018). Subsequently, the first-in-human phase I clinical trial of ApTOLL was performed in healthy volunteers and showed good pharmacokinetic and safety profiles (Aliena-Valero et al., 2024). Another Phase Ib/IIa clinical study (APRIL) is in progress to evaluate the administration of ApTOLL together with endovascular treatment (in acute ischemic stroke (AIS) patients, promoting its future clinical positioning for stroke therapy (Hernandez-Jimenez et al., 2022). In fact, a highly specific aptamer, L1, for the human soluble growth-stimulating gene protein (sST2) was *in vitro* developed in our latest study (Hernandez-Jimenez et al., 2023). The IL-33/ST2 pathway plays a critical role in neuroinflammation-related CNS diseases, including ischemic stroke. As a decoy receptor of IL-33, specific targeting of sST2 may be a rational choice to attenuate neuroinflammation and promote CNS repair (Ayass et al., 2023). Aptamers against sST2 may be an available approach for detecting sST2 concentrations and restoring the protective effects of IL-33/ST2 against ischemic stroke.

**TABLE 2:** Summary of aptamers targeting inflammatory responses for stroke treatment and molecular detection.

Aptamer	Mode of action	Application	Developmental stage
CRP-apt	Binds to CRP	Patented CRP aptamers have been developed as biomolecules used for detection or experimental assays with the purpose of CRP inhibition (Miramontes-Espino and Romero-Prado, 2013)	Preclinical (*in vitro &* *in vivo*)
NOX-D20	Binds to mouse C5a and C5a-desArg	Blocks the binding of C5a to CD88 (Yatime et al., 2015)	Preclinical (*in vitro*)
aC5a-FNA	A platform for delivering aC5a	Selectively reduces C5a-mediated neurotoxicity and effectively alleviates inflammation and oxidative stress in the ischemic brain (Li et al., 2019)	Preclinical (*in vitro &* *in vivo*)
ApTOLL	A truncated form TLR4-blocking DNA aptamer ApTLR#4FT	Shows a long-lasting protective effect against brain injury induced by MCAO in rat (Fernandez et al., 2018; Aliena-Valero et al., 2024) A very good pharmacokinetic and safety profile in healthy volunteers (Hernandez-Jimenez et al., 2022) In progress to evaluate the administration of ApTOLL together with EVT in patients with AIS (Hernandez-Jimenez et al., 2023)	Preclinical (*in vitro &* *in vivo*) Phase I Phase Ib/IIa
Apt-L1	Specific target to human sST2 (Zeng et al., 2024)	/	/

Note: CRP, C-reactive protein; C5a, complement component 5a; aC5a, anti-C5a aptamers; FNA, framework nucleic acid; TLR4, Toll-like receptor 4; MCAO, middle cerebral artery occlusion; EVT, endovascular treatment; AIS, acute ischemic stroke; sST2, soluble growth stimulating gene protein.

### 3.3 Target to specific biomarkers in key cells

Cell-specific targeted therapies for ischemic stroke are currently under development. Superior to cell-SELEX, brain slice-based SELEX allows for the evolution of aptamers for more target molecules in various cells. Vigilin exhibits enhanced release from cultured hippocampal neurons after *in vitro* oxygen glucose deprivation. Using frozen brain slices from a mouse model of ischemia, a specific aptamer LCW17 targeting vigilin was selected to be potentially applied to define the molecular mechanism and diagnosis underlying ischemic stroke (Zeng et al., 2024). Regulator of calcineurin 1 isoform 1 (RCAN1.1) is highly expressed in the brain (Liu et al., 2019). Overexpression of RCAN1.1 significantly increased cell apoptosis in a mouse middle cerebral artery occlusion model and in cultured neurons under oxygen glucose deprivation conditions. The RNA aptamer of RCAN1.1, R1SR13, was recently shown to attenuate RCAN1.1-induced neuronal apoptosis, both *in vivo* and *in vitro*, providing a potential approach for AIS prevention (Peiris and Keating, 2018). Cerebrovascular endothelial cells (CECs) are integral components of the BBB and are the first-line brain cells affected by cerebral ischemia. Vascular cell adhesion molecule-1 (VCAM-1) expressed on endothelial cells can regulate vascular adhesion, leukocyte migration, and inflammatory infiltration (Yun et al., 2022; Supanc et al., 2011). Since the expression of VCAM-1 was increased in CECs after stroke, a VCAM-1-based RNA aptamer was recently obtained, and an effective aptamer-based delivery platform to target CECs was constructed to treat cerebral ischemia (Franx et al., 2021; Chauveau et al., 2007). Several cell types such as astrocytes, microglia, neutrophils, and macrophages also play critical roles in tissue damage and repair, meriting further investigation based on aptamer technology.

### 3.4 Other aptamer applications in ischemic stroke

Other aptamer-based applications have also been reported to be promising in stroke research. High levels of homocysteine (Hcy) are associated with an increased risk of many diseases, including stroke (Hu et al., 2023). In a recent study, a DNA aptamer (apt8) that binds to the alkane thiol chain of Hcy with exceptional specificity against cysteine was successfully isolated; this specific aptamer combined with a reusable fluorescent optical fiber biosensor greatly expanded the practical utility of aptasensors in the molecular diagnosis of ischemic stroke (Pinzon et al., 2023). Similarly, a one-step aptasensor based on multifunctional carbon nanotubes using square-wave voltammetry, and a label-free aptasensor based on gold nanoparticles were also developed to realize the ultrasensitive detection of Hcy (Zhou et al., 2023; Chen et al., 2023). In addition to molecular diagnosis, aptamers can serve as molecular imaging probes for the rapid detection of thrombi. The aptamer Tog25t targeting thrombin can rapidly localize to visualize pre-existing clots *in vivo*, which may be applied in acute care and perioperative settings for stroke (Beitollahi et al., 2020). Likewise, an aptamer-based expansion microscopy platform was recently developed for the super-resolution imaging of neuronal dendritic spines. Among these, the aptamer yly12 could specifically bind to the L1 cell adhesion molecule, a transmembrane protein expressed on neurites. A methacryloyl moiety was conjugated to the 5ʹ end of yly12 to achieve physical magnification of dendritic spine morphology, which contributed to its application in elucidating pathological mechanism of early stroke (Gray et al., 2023). Moreover, a novel aptamer-based proteomic assay (SOMAscan) has facilitated the unbiased screening of outstanding proteins in the cerebrospinal fluid and plasma samples from ischemic animals or AIS patients, providing an extended strategy to support future studies in this field (Zhuo et al., 2023; Simats et al., 2018).

## 4 Conclusion and future perspective

**FIGURE 2:**
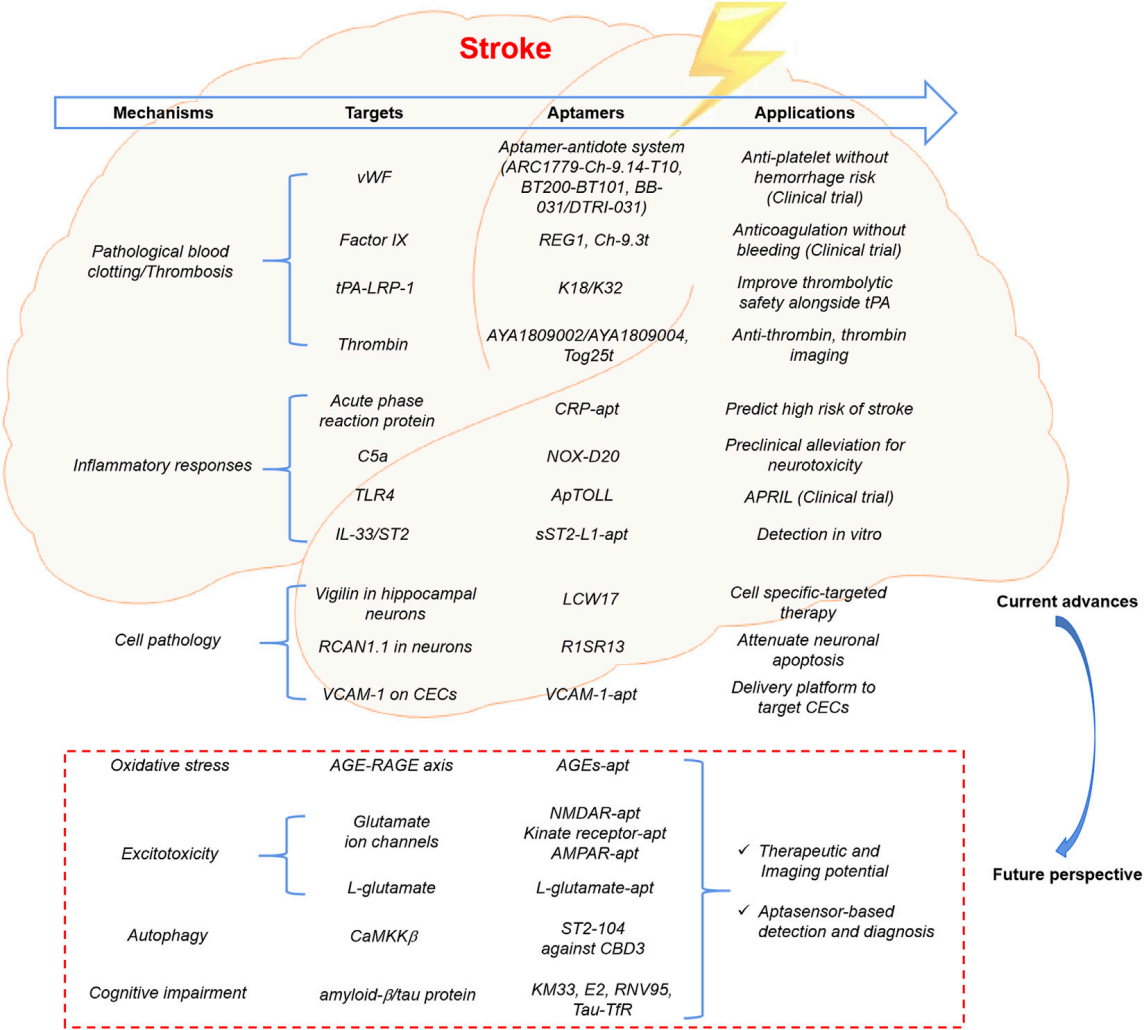
Current advances and future perspective of aptamer applications in ischemic stroke. Aptamers have gradually become a useful tool for targeted therapy and biomarker discovery in the field of ischemic stroke, via targeting to pathological blood clotting or thrombosis, inflammatory responses, and specific biomarkers in the key cells. Capture or function blocking via small molecule-binding aptamers targeting the important pathological mechanisms would have tremendous potential in the future research and applications, such as oxidative stress, excitotoxicity, autophagy, and cognitive impairment.

It is possible that SOMAscan can be an effective tool for screening promising candidates in the cerebrospinal fluid, peripheral circulation, and even brain tissue, which contributes to the further elucidation of the molecular mechanism of cerebral ischemia. Subsequently, a novel diagnostic and treatment strategy can be developed, in which the screening of specific aptamers is also viable. According to the reported SOMAscan results, several candidate proteins have been identified, including creatine kinase B-type, amphiregulin, pyridoxal phosphate phosphatase, cytidine monophosphate kinase, and calcium/calmodulin-dependent protein kinase II (CaMK2) members, and the role of these and related pathways should be further explored to identify possible therapeutic targets (Zhuo et al., 2023; Simats et al., 2018). In addition to pathological blood clotting and thrombosis, neuroinflammation, and apoptosis, the pathological mechanisms underlying stroke involve other complex processes, such as oxidative stress, excitotoxicity, and autophagy (Mao et al., 2022). Capture or functional blocking via small molecule-binding aptamers targeting these mechanisms may have tremendous potential in the field of stroke (Figure 2). i) The formation and accumulation of advanced glycation end products (AGEs) can evoke oxidative stress and inflammatory responses by interacting with a receptor for AGEs (RAGE), contributing to the development of various diabetes- or aging-associated disorders (Pala et al., 2024). Therefore, the therapeutic potential of DNA aptamers against the AGE-RAGE axis in diabetes-related complications is promising, particularly when applied to stroke (Mao et al., 2022; Rao et al., 2022). ii) In several neurological disorders, excessive activation of glutamate ion channels, including N-methyl-d-aspartate (NMDA), α-amino-3-hydroxy-5-methyl-4-isoxazole, and kainate receptors, is involved in excitotoxicity. 2′F-modified RNA aptamers against GluN2A-containing N-methyl-d-aspartate, 2ʹ-fluoro-modified kinate receptor-selective RNA aptamers, and chemically modified α-amino-3-hydroxy-5-methyl-4-isoxazole receptor RNA aptamers have been designed, which may be used in targeted drug delivery, imaging, or therapeutic intervention for stroke (Yamagishi et al., 2016; Yamagishi and Matsui, 2018; Lee et al., 2014). Additionally, a peptide aptamer against L-glutamate-based electrochemical amperometric sensors has been developed as a novel detection tool (Huang et al., 2017). iii) ST2-104, a non-arginine (R9)-fused Ca^2+^ channel-binding domain 3 peptide aptamer, attenuates neuronal apoptosis by inhibiting CaMK kinase β-mediated autophagy, providing novel insights into the potential neuroprotective effects of ST2-104 in cerebral ischemia (Jaremko et al., 2020). Moreover, cognitive impairment and dementia are major sources of morbidity and mortality after stroke; therefore, the development of precise sensing tools for early prevention and diagnosis of related complications is essential (Wang et al., 2022). Recently, aptamer-based biosensors and novel test kits have been designed to quantitatively monitor cognitive impairment-associated biomarkers, such as classical amyloid−β peptides and tau proteins, offering a promising means for application in cerebral ischemia (Yao et al., 2021).

Aptamer-based diagnostic strategies targeting specific biomarkers have gradually been applied to ischemic stroke. Generally, biosensors comprise three key components: molecular recognition, signal conversion, and signal output. When developing an aptamer-based biosensor, the molecular elements of the aptamer are vital to determine its ability to achieve sensitive and rapid detection. Combined with novel biomaterials, the common diagnostic platforms based on aptasensors and their principles are presented in Supplementary Figure S1 and are expected to replace enzyme-linked immunoreactions and detect specific biomarkers for stroke prevention and diagnosis (Rost et al., 2022; Zamanian et al., 2022; Verma et al., 2023; Feagin et al., 2018; Nooranian et al., 2022; Ueno, 2021; Scognamiglio et al., 2015; Dong et al., 2020). Considering the advantages and disadvantages of aptamers, antibody-aptamer hybrid biosensors and multi-target aptasensors were recently designed to optimize the detection capacity for disease diagnosis (Buranachai et al., 2012; Xu and Lu, 2010). Furthermore, diverse modifications of aptamers would offer a greater chance of success for therapeutic and diagnostic applications in ischemic stroke, including aptamer-drug conjugates, targeted delivery materials, therapeutic agents, and molecular imaging (Jarczewska and Malinowska, 2020). For example, aptamer-siRNA chimeras for nerve cell-targeted delivery of therapeutic oligonucleotides may be further highlighted for clinical development in the field of stroke (Nguyen et al., 2016; Ni et al., 2021; Kruspe and Giangrande, 2017). Overall, along with the advancements in nanotechnology, microfluidics, and microarrays, aptamers may play an important role in clinical applications.
