# Supplementary material for: The prospective approach for aptamers applied in the treatment and molecular diagnostics of ischemic stroke
Source: Front Pharmacol. 2025 Apr 28;16:1553337. doi: 10.3389/fphar.2025.1553337 (PMC12079141; doi:10.3389/fphar.2025.1553337)
Supplement: Supplementary file 1 [file DataSheet1.pdf]

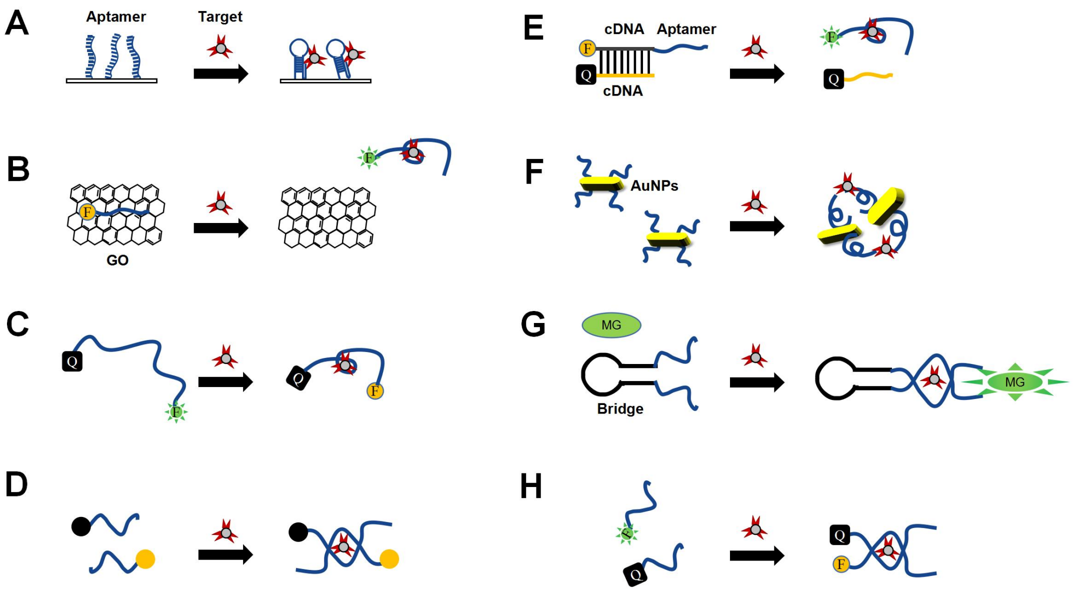

Supplementary Figure 1. The reported diagnostic platforms based on aptasensors. (A) Simple binding-based platform using aptamers instead of antibodies in ELISA, immunoprecipitation and so on. (B) Graphene oxide (GO)-based platform adsorbing the aptamer linked with a fluorescent probe for detection. (C) Fluorescence resonance energy transfer (FRET)-based platform. Aptamer-target binding can change the fluorescence intensity. (D) Nanoparticle assembly induced by split aptamer-target binding. (E) Structure-switching signaling aptamer-based platform via the cDNA linking a quencher (Q) or fluorophore (F). (F) Detection based on gold nanoparticles (AuNPs) assembly induced by aptamer-target binding. (G) Aptasensor based on nucleic acid hybridization (the aptamer hybridized with bridge chain). The hybridization can prevent binding of malachite green (MG) to aptamer, which can be reversed in the presence of target. (H) Self-assembly of split aptamers in the presence of target molecules.
